# Termite Resistance of MDF Panels Treated with Various Boron Compounds

**DOI:** 10.3390/ijms10062789

**Published:** 2009-06-19

**Authors:** Mustafa Usta, Derya Ustaomer, Saip Nami Kartal, Sedat Ondaral

**Affiliations:** 1 Karadeniz Technical University, Faculty of Forestry, Trabzon, Turkey; E-Mails: usta@ktu.edu.tr (M.U.); ondaral@ktu.edu.tr (S.O.); 2 Istanbul University, Faculty of Forestry, Istanbul, Turkey; E-Mail: snkartal@istanbul.edu.tr (S.N.K.)

**Keywords:** medium density fiberboard (MDF), termite resistance, borax, boric acid, zinc borate, sodium perborate tetrahydrate, *Coptotermes formosanus* Shiraki

## Abstract

In this study, the effects of various boron compounds on the termite resistance of MDF panels were evaluated. Either borax (BX), boric acid (BA), zinc borate (ZB), or sodium perborate tetrahydrate (SPT) were added to urea-formaldehyde (UF) resin at target contents of 1%, 1.5%, 2% and 2.5% based on dry fiber weight. The panels were then manufactured using 12% urea-formaldehyde resin and 1% NH_4_Cl. MDF samples from the panels were tested against the subterranean termites, *Coptotermes formosanus* Shiraki. Laboratory termite resistance tests showed that all samples containing boron compounds had greater resistance against termite attack compared to untreated MDF samples. At the second and third weeks of exposure, nearly 100% termite mortalities were recorded in all boron compound treated samples. The highest termite mortalities were determined in the samples with either BA or BX. Also, it was found that SPT showed notable performance on the termite mortality. As chemical loadings increased, termite mortalities increased, and at the same time the weight losses of the samples decreased.

## 1. Introduction

Medium density fiberboard (MDF) is one of the most important wood based panels. It is widely used as a structural material for furniture and building construction. It is desirable to use resistant products for these applications. Wood based panel companies are especially interested in treating products with protective chemicals that prevent biological damage [1]. Although MDF is an ideal material for some applications, it is also prone to biological degradation like the wood and other wood based panels. Wood can easily deteriorate because of a variety of organisms such as decay fungi and insects [2]. Chung *et al*. reported that wood based panels were as susceptible to microorganisms as solid wood [3]. Therefore, it is important to increase the resistance of wood and wood based panels to biological attacks by using various protective chemicals. A variety of protective chemicals have been used for this purpose in the wood protection industry, but many of these chemicals are not preferred due to either high costs, low efficacy, corrosiveness or chemical toxicity for environment and health [4]. In the recent years, boron compounds are widely used as important wood protective chemicals because they do not have these unfavorable properties. They are odorless, colorless, non-flammable, non-corrosive, cost-effective, easily applicable, and biologically active [4–6]. Besides, these compounds have low mammalian toxicity and low environmental damage. It is reported that boron compounds effectively provide protection wood from termite, decay fungi etc., under non-leaching conditions [7–9].

The Formosan subterranean termite (*Coptotermes formosanus* Shiraki) is the most economically important and structually destructive insect pest in the some states, especially in Hawaii. They attack above-ground wood structures and can remain above ground. In this way, they cause considerable damages to wood based materials. Therefore, preventing the damages caused by these termites has a great importance for homeowners and commercial builders. Recently, some methods such as physical and chemical barriers, termite baits, soil insecticide applications and use of treated wood with various protective chemicals have been applied to prevent the termite damage [10–12]. In this context, boron compounds are extensively used to provide termite control. For the purpose of protecting wood and wood based panels against termite attacks, several studies have focused on boron compounds. Previous studies have strongly supported the usability of boron compounds such as zinc borate, boric acid, disodium octaborate tetrahydrate, etc., for the treatment of wood and wood based panels. These studies also stated that boron compounds provided good protection against termites and decay fungi and are notably toxic against insects [4,5,7–9,11–18].

In this study, MDF samples from panels treated with boric acid, borax, zinc borate, or sodium perborate tetrahydrate, together with untreated MDF samples, were exposed to subterranean termites under laboratory conditions. The objective of this study was to evaluate the efficacy of all these chemicals towards termite attack and to compare the effects of SPT, which has been extensively used as a bleaching agent.

## 2. Experimental

### 2.1. Chemicals

Boric acid (H_3_BO_3_, BA), borax (Na_2_B_4_O_7_·10H_2_0, BX), zinc borate (2 ZnO·3B_2_O_3_·3.5H_2_O, ZB), and sodium perborate tetrahydrate (NaBO_3_·4H_2_O, SPT) were used as chemical agents in the production of panels. BX, BA and SPT were obtained from ETIBOR Company (Turkey) and ZB was purchased from Riedel-de Haën (Germany).

### 2.2. Manufacture of MDF Panels

MDF panels were manufactured from commercially produced fibers. The fibers were supplied by the ÇAMSAN Company in Ordu, Turkey. Before the panel production, the fibers were dried in a laboratory oven until they reached approximately 2–3% moisture content. All chemicals were used at the concentrations of 1%, 1.5%, 2% and 2.5% based on oven dry fiber weight. Urea-formaldehyde (UF) was used as adhesive at the ratio of 12% based on oven dry fiber weight and NH_4_Cl was used at a 1% ratio to furnish weight as hardener. Before applications, the chemical solutions were prepared and mixed into the adhesive. The mixtures with UF were sprayed onto the fibers before the mats were manually formed. The mats were pressed using a computer control press. The selected pressing conditions were 30 kg/cm^2^, 180 °C and 6 minutes. The MDF panels were conditioned in a climatized room at 22 °C and of 65% relative humidity until they reached equilibrium moisture content.

### 2.3. Laboratory Termite Resistance Tests

**Figure 1.**
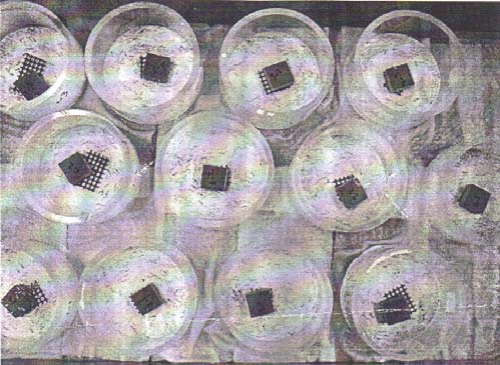
Assembled containers and tests samples for termite tests.

Untreated and treated MDF samples were exposed to the subterranean termites, *Coptotermes formosanus* Shiraki, according to the JIS K 1571 standard method [19]. An acrylic cylinder (80 mm in diameter, 60 mm in height) whose lower end was sealed with a 5 mm thick hard plaster (GC New Plastone, Dental Stone, G-C Dental Industrial Corp., Tokyo, Japan) was used as a container. A test specimen was placed at the centre of the plaster bottom of the test container. A total of 150 worker termites collected from a laboratory colony maintained by RISH, Kyoto University, Kyoto (Japan) were introduced into each test container together with 15 termite soldiers (Figure 1). Five samples per treatment were assayed against the termites. The assembled containers were set on damp cotton pads to supply water to the samples and kept at 28°C and >85% RH in darkness for three weeks. The mass losses of the samples due to termite attack were calculated based on the differences in the initial and final oven-dry (60 °C, 3 days) weights of the samples after cleaning off the debris from the termite attack.

## 3. Results and Discussion

Average weight losses, standard deviation, p-values and homogeneity groups were analyzed by ANOVA and Duncan tests for each boron compound and are given in Table 1. Boric Acid Equivalent (BAE) values were also calculated for each compound and concentrations and are shown in Table 1. Univariate variance analysis was also done to determine interaction between chemical concentration and boron chemicals in the weight losses of MDF samples. This is shown in Table 2.

As can be seen in Table 1, treatments of MDF panels with BA, ZB, SPT or BX resulted in decreased weight losses when compared to control MDF samples. It is clearly seen that the average weight loss of control samples was 14.17%, while the highest average weight loss values obtained from samples treated with ZB and SPT at 1% concentration were 12.80 % and 12.67%, respectively. It was found that the lowest average weight loss values were obtained from samples treated with BA (7.51%) and BX (8.60%). Also, it was clear that BA and BX were much more effective than ZB and SPT at the lowest chemical concentration (1%). The efficacy of SPT and ZB in decreasing weight losses started to be noticeable at 1.5% concentration.

It was observed that increasing chemical concentrations reduced the weight loss of samples treated with all chemicals. Also, previous studies reported that increasing boron compound retention levels reduced weight loss of treated samples for the termite resistance test [12,13,20,21].They also reported that the samples treated with various boron compounds were consumed significantly less than the control samples. The efficacy of boron compounds and concentrations was evaluated by analysis of variance and the results are shown in Table 2. Regarding the results, the boron compounds and concentrations, and the interactions were significant in the weight losses of MDF samples.

According to our results it is possible to say that all chemicals were effective on the weight loss and provided structural protection. The treated MDF samples were found almost intact after termite exposure at the end of week 3. The termite mortality (%) of the MDF samples is shown in Table 3. It was found that termite mortality was greater for all samples with boron compounds in comparison with the control MDF samples. At the end of the first week, no mortality was observed in the samples with BA, ZB, or SPT; however, a remarkable effect was observed in the samples with all concentrations of BX. This effect increased as the concentration of BX increased. In the second week of exposure, no termite mortality was seen in control MDF samples, while considerable mortality was recorded for MDF samples produced with all types of the chemicals. Termite mortality was found as 49.8%, 20.8%, 15.5%, 0% for the samples with BX, BA, ZB, SPT, respectively, at the 1% concentration level. The mortality rate reached to 100% at 1.5% concentration level of BA and SPT, and mortality rates of 78% and 85% at 1.5% and 2% concentration levels, respectively, were achieved for ZB and BX-treated MDF samples.

At the end of the exposure period, complete termite mortalities were recorded in all samples. While the termite mortality rates at 1% concentration were 100%, 88.80%, 56.70, 37.90% for the samples with BX, BA, SPT, ZB, respectively, the mortality rate reached 100% at 1.5%, 2% and 2.5% concentration levels and the chemicals provided excellent protection against termite attack. Slight termite mortality was recorded for control MDF samples at the end of three weeks. This value for the control MDF samples was found to be 18.80%. According to this value and visual observations almost no termite death occurred on the control samples. The samples with BX, BA, or SPT were less attacked by the termites than the samples with ZB. This may be possibly due to the various reasons such as toxic properties and efficacy of these chemicals. As can be seen Table 3, It is possible to say that 1.5% concentrations of all chemicals were sufficent to provide almost 100% termite mortality at the end of exposure period. In our study, high mortality rates were also obtained with samples treated with SPT, which is one of the chemicals especially used in safety bleach formulations, detergents and tooth powders [22]. Furthermore, it is seen from Table 3 that termite mortality rate increased as exposure time and chemical concentrations increased.

Concentrations, boron compound types and concentrations-boron compound types were evaluated by the analysis of repeated measure variance for termite mortality and the results are shown in Table 4. Regarding the results, concentrations, boron compound types and concentration-boron compound types were found significant in termite mortality. It can be concluded from these results all chemicals provided a strong effect on the termite mortality.

Our results in this study were similar to and in accordance with the results obtained from previous studies demonstrating effects of boron compounds against termite attacks [12,13,15–18,20,21,23,24].

## 4. Conclusions

In this study, the termite resistance of MDF panels produced using various boron compounds was evaluated. It was found that all four types MDF samples produced with BA, ZB, SPT or BX had high resistance against termite attack when compared to control MDF samples and all chemicals provided remarkable effects on the termite mortality and weight losses. At the end of three weeks, effective protection was obtained against termites and 100% termite mortality was recorded due to the toxic effects of the chemicals. Furthermore, weight loss in the samples containing the chemicals remarkably decreased when compared to weight loss of control MDF samples. Although all chemicals had high efficiency on termite mortality, the efficacy of BX started in the first week of the exposure. While the termite mortality remarkable increased, the weight loss reduced depends on increasing concentrations of chemicals. Based on the findings in the study, it might be concluded that the chemicals at 1.5% concentration provide nearly 100% termite mortality at the end of exposure time. Furthermore, it is found that SPT showed a strong ability to kill termites, as much as BA and BX. It is likely that all boron compounds used in the study could prevent attack by *Coptotermes formosanus* and cause complete termite mortality.

## Figures and Tables

**Table 1.** ANOVA Analysis and Duncan test results of weight losses in treated MDF samples by various boron compounds after exposure to termite for three weeks.

**Samples**	BAE* (%)	**X-means**	**Standard Deviation**	**Homogeneity Groups**	***p*-values**
1% BA	1	7.51	0.43	D	0.000
1.5% BA	1.5	4.44	0.12	C	
2% BA	2	5.82	0.14	A	
2.5% BA	2.5	5.08	0.19	B	
*Control*	–	*14.17*	*0.73*	*E*	

1% ZB	1.17	12.80	2.22	B	0.000
1.5 % ZB	1.76	4.34	0.05	A	
2 % ZB	2.34	4.29	0.17	A	
2.5 % ZB	2.93	4.94	1.06	A	
*Control*	–	*14.17*	*0.73*	*C*	

1 % SPT	2.49	12.67	1.48	C	0.000
1.5% SPT	3.74	5.56	0.77	B	
2 % SPT	4.98	5.52	0.40	B	
2.5% SPT	6.23	4.26	0.75	A	
*Control*	–	*14.17*	*0.73*	*D*	

1% BX	1.5	8.60	1.58	A	0.001
1.5 % BX	2.25	6.71	1.18	A	
2 % BX	3	8.12	5.70	A	
2.5 % BX	3.75	7.48	0.33	A	
*Control*	–	*14.17*	*0.73*	*B*	

* **BAE:** Boric Acid Equivalent values.

**Table 2.** Analysis of univariate variance for weight losses of treated MDF samples after exposure to termite for three weeks.

**Source**	**Type III Sum of Squares**	**df**	**Mean Square**	**F**	**Sig.**
Intercept	5447.142	1	5447.14	1977.05	0.000
Concentrations(A)	429.022	3	143.01	51.91	0.000
Boron Compound Types(B)	51.075	3	17.03	6.18	0.001
Interaction(A*B)	187.841	9	20.87	7.58	0.000
Error	234.191	85	2.76		

**Table 3.** Termite mortality of MDF samples produced using 1%, 1.5%, 2%, 2.5% BA, ZB, SPT and BX.

**Termite Mortality (%)**			
**Samples**	**Week 1**	**Week 2**	**Week 3**
1% BA	0.00	20.80	88.80
1.5% BA	0.00	100.00	100.00
2% BA	0.00	100.00	100.00
2.5% BA	0.00	100.00	100.00

1% ZB	0.00	15.50	37.90
1.5 % ZB	0.00	78.00	83.03
2 % ZB	0.00	85.00	100.00
2.5 % ZB	0.00	85.00	100.00

1 % SPT	0.00	0.00	56.70
1.5% SPT	0.00	100.00	100.00
2 % SPT	0.00	100.00	100.00
2.5% SPT	0.00	100.00	100.00

1% BX	20.80	49.80	100.00
1.5 % BX	27.60	78.00	100.00
2 % BX	49.80	85.00	100.00
2.5 % BX	62.70	85.00	100.00

Control	0	0	18.80

**Table 4.** Analysis of repeated measure variance for termite mortality of treated MDF samples after exposure to termite for each weeks.

**Source**	**Type III Sum of Squares**	**df**	**Mean Square**	**F**	**Sig.**
Intercept	328521.56	1	328521.56	66767.33	0.000
Concentrations(A)	33073.51	3	11024.50	2240.57	0.000
Boron Compound Types(B)	10121.14	3	3373.72	685.66	0.000
Interaction(A*B)	3820.32	9	424.48	86.27	0.000
Error	167.29	34	4.92		

## References

[1] Barnes HM Murphy RJ Effect of vapor boron treatment on some properties of wood strand and fiber composites Compos. Part A-Appl. Sci. Manuf 2006 37 1402 1405

[2] Morrell JJ Wood based building components: What have we learned? Int. Biodeterior. Biodegradation 2002 49 253 258

[3] Chung WY Wi SG Bae HJ Park BD Microscopic observation of wood-based composites exposed to fungal deterioration J. Wood Sci 1999 45 64 68

[4] Yalinkilic MK Improvement of boron immobility in the borate treated wood and composite materialsPhD ThesisKyoto University Japan 2000

[5] Kartal SN Yoshimura T Imamura Y Decay and termite resistance of boron-treated and chemically modified wood by in situ co-polymerization of allyl glycidyl ether (AGE) with methyl methacrylate(MMA) Int. Biodeterior. Biodegradation 2004 53 111 117

[6] Baysal E Yalinkilic MK A new boron impregnation technique of wood by vapor boron of boric acid to reduce leaching boron from wood Wood Sci. Technol 2005 39 187 198

[7] Wong AHH Grace JK Laboratory Evaluation of the Formosan subterranean termite resistance of borate–treated rubberwood chipboard The International Research Group on Wood Preservation, 35th Annual meeting, IRG/WP 04-30359 Ljubljana, Slovenia 6–10 June 2004

[8] Drysdale JA Boron treatments for the preservation of wood-a review of efficacy data for fungi and termites The International Research Group on Wood Preservation, 25th Annual meeting, IRG/ WP 94-30037 Bali, Indonesia 29 May–3 June 1994

[9] Grace JK Review of recent research on the use of borates for termite prevention Proc. of the 2nd International Conference on Wood Protection with Diffusible Preservatives and Pesticides. Forest Prod. Soc. Madison, WI., USA 1997

[10] Grace JK Woodrow RJ Yates JR Distribution and management of termites in Hawaii Sociobiology 2002 40 87 93

[11] Gentz MC Grace JK The response and recovery of the Formosan subterranean termite (*Coptotermes formosanus* Shiraki) from sublethal boron exposures Int. J. Pest Manag 2009 55 63 67

[12] Lee S Wu Q Smith WR Formosan subterranean termite resistance of borate-modified strandboard manufactured from southern wood species: a laboratory trial Wood Fiber Sci 2004 36 107 118

[13] Tsunoda K Watanabe H Fukuda K Hagio K Effects of zinc borate on the properties of medium density fiberboard Forest Prod. J 2002 52 62 65

[14] Tsunoda K Preservative properties of vapor-boron-treated wood and wood-based composites J. Wood Sci 2001 47 149 153

[15] Kartal SN Ayrilmis N Blockboard with boron-treated veneers: Laboratory decay and termite resistance tests Int. Biodeterior. Biodegradation 2005 55 93 98

[16] Kartal SN Ayrilmis N Imamura Y Decay and termite resistance of plywood treated with various fire retardants Build. Environ 2007 42 1207 1211

[17] Grace JK Yamamota RT Tamashiro M Resistance of borate-treated Douglas-fir to the Formosan subterranean termite Forest Prod. J 1992 42 61 65

[18] Gentz MC Grace JK Different boron compounds elicit similar responses in Coptotermes formosanus (Isoptera: Rhinotermitidae) Sociobiology 2007 50 633 641

[19] *JIS K 1571*, Test methods for determining the effectiveness of wood preservatives and their performance requirements (in Japanese), Japanese Standard Association, 2004.

[20] Akbulut T Kartal SN Green F III Fiberboards treated with *N’-N*-(1,8-Naphtyhalyl) hydroxylamine (NHA-Na), borax, and boric acid Forest Prod. J 2004 54 59 64

[21] Ahmed BM French JR Vinden P Evaluation of borate formulations as wood preservatives to control subterranean termites in Australia Holzforschung 2004 58 446 454

[22] Mandare PN Pangarker VG Semi-batch reactive crystallization of sodium perborate tetrahydrate:effect of mixing parameters on crystal size Chem. Eng. Sci 2003 58 1125 1133

[23] Kartal SN Burdsall HH Green F III Accidental mold/termite testing of high density fiberboard (HDF) treated with borates and N’N-naphthaloylhydroxylamine(NHA) The International Research Group on Wood Preservation, 34th annual meeting, IRG/WP 03-10462 Brisbane, Queensland, Australia 18–24 May 2003

[24] Ayrilmis N Kartal SN Laufenberg TL Winandy JE White RH Physical and mechanical properties and fire, decay, and termite resistance of treated oriented strandboard Forest Prod. J 2005 55 74 81

